# Physiochemical Analysis of Drinking Water and Treatment with a Homemade Filter: A Case Study of Illu Abba Bor Zone, Ethiopia

**DOI:** 10.1155/2022/4333938

**Published:** 2022-12-31

**Authors:** Badhane Gudeta, M. Venkata Ratnam, Raja Mohan

**Affiliations:** Department of Chemical Engineering, Mettu University, Metu, Ethiopia

## Abstract

The drinking water quality was evaluated in order to provide a continuous supply of clean and safe drinking water for the preservation of public health. The study area consists of three villages: Tulube, Seddo, and Serdo, all of which are located near Mettu town, which is about 550 kilometers south-west of Ethiopia's capital, Addis Ababa. The physical and chemical parameters of the collected drinking water samples were assessed, including pH, turbidity, conductivity, total suspended solids (TSS), total dissolved solids (TDS), and the presence of heavy metals. The samples were examined in the laboratory, and the findings were compared to the World Health Organization (WHO) standards. Almost all of the physiochemical indicators were safe and within the permissible limit for drinking water quality. However, lead ion concentrations were found to be above the WHO standards. An adsorbent produced from banana pseudostems was used to remove lead ions from drinking water. The equilibrium parameters were determined using the Langmuir adsorption isotherm. The drinking water was treated for 4 h in a homemade adsorption column composed of filter medium (sand, charcoal, and powder of treated banana pseudostem). The data revealed that lead ions removal was nearly 70%, but still above the WHO standards.

## 1. Introduction

The oceans contain the majority of the world's water, which is unsuitable for human consumption. Only around 3% of the world's water is thought to be fresh, with 2.97% preserved in glaciers and ice caps. The remaining 0.03% is collected in the form of surface and ground water for human use. A sufficient quantity of clean drinking water that is free of contamination is a key necessity for a sustainable human life [1, 2]. Disease-causing microbes and chemical contaminants that are hazardous to one's health should not be present in drinking water. It should not be cloudy, high, or muddy, and it should adhere to the WHO guidelines. It should also be free of contaminants such as organic and inorganic pollutants, heavy metals, and pesticides [3]. It is critical that all people, particularly those living in developing nations, have access to safe, affordable drinking water [4]. Human activities such as urbanization, industrialization, agricultural activity, and other sorts of human activity have all contributed to an increase in the contamination of surface and ground water in recent decades [2–5]. Modernization, along with other human activity, continues to be a role in the transfer of waterborne diseases and has an impact on aquatic life [6, 7]. Furthermore, it has the capacity to spread diseases in countries especially in the poor countries. Concerns about human health caused by waterborne infections are especially widespread in less developed nations such as Ethiopia, which lacks access to clean water of sufficient quality for human use. The World Health Organization (WHO) estimates that polluted drinking water accounts for roughly 94% of the worldwide diarrheal sickness and 10% of the total disease burden [5–7]. Hence, access to safe drinking water is becoming increasingly crucial. Water bodies are commonly thought to be a source of metal buildup in aquatic animals, which can have long-term repercussions on both human health and the ecosystems in which they reside [7]. Researchers are currently investigating the presence of heavy metals in drinking water from both natural and anthropogenic sources [8–10]. Heavy metals have found their way into both surface and ground water. The pervasiveness, toxicity, and possible harm to human health presented by these metals all have an impact on water quality [11, 12]. Lead, zinc, copper, arsenic, cadmium, chromium, nickel, and uranium, as well as mercury, are heavy metals that cause the most concern. The lead concentration in drinking water is a major concern. Even in low amounts, Pb can impair adults' and children's neurological systems. When lead-containing plumbing components decay in acidic or low-mineral water, lead can enter drinking water. Lead service lines are a significant source of lead [13].

Researchers across the world conducted studies to assess the drinking water quality. An experiment was carried out to see how a homemade filter affected turbidity, fluoride, pH, and temperature [2]. According to the study, filtering water significantly decreased physical, chemical, and biological pollutants. The filtering device is small and light, making it ideal for travel. A comparative chemical study assessed the physical, chemical, and biological features of an Egyptian drinking water treatment plant [4]. The evaluation includes fresh water from the canal inflow, drinking water, and sand filter backwashing water. This report contains various ideas for policymakers that can save 20% of wasted water while also protecting canal waterways. To protect public health, the drinking water quality in Malaysia's Perak state was investigated [5]. Drinking water samples from residential and commercial regions around the state were tested for pH, turbidity, conductivity, TSS, TDS, and heavy metals in this context. The values of the parameters were compared to the WHO standards as well as local criteria such as the National Drinking Water Quality Standard (NDWQS). Each metric satisfied the WHO and NDWQS requirements.

In Ethiopia, wells and springs are the primary sources of drinking water, supplying large urban and rural areas. Despite the fact that the government lacks regular and thorough water quality testing programs, there are rising indicators of water pollution concerns in some areas. Soil erosion, residential trash from urban and rural regions, and industrial wastes might all be important sources of pollution. There is not much adequate research done on drinking water standards and heavy metal pollution in the country. Endale et al. investigated Pb amounts in drinking water in Addis Ababa, Ethiopia's capital city. According to the study, Addis Ababa's drinking water is likely to be a source of lead exposure [13]. Alemu et al. [14] conducted a retrospective study on physicochemical quality of drinking water sources of Ethiopia. The study used 983 water samples collected from different regions of the country for testing. Very high sodium and chloride concentrations were recorded in spring, tap, and well water sources of the region such as Somali, Afar, and Oromia. The research was conducted by Mebrahtu and Zerabruk [15] to analyze the state of drinking water quality in the Tigray region of northern Ethiopia. The primary goal of this paper is to determine the levels/concentrations of some physicochemical parameters and heavy metals and to compare the values with the national and international organization (such as WHO) recommended drinking water standards. The results demonstrate that certain samples' electrical conductivity (EC), total dissolved solid (TDS), turbidity, and concentrations of various heavy metals (As, Cd, Cr, Fe, Ni, and Pb) are greater than the WHO standards.

Heavy metal removal can be achieved using a number of methods, including chemical precipitation, ion exchange, chemical oxidation, reduction, reverse osmosis, ultrafiltration, electro dialysis, and adsorption [16–18]. The great majority of them are traditional and pricey. Traditional water purifying technologies may be excessively expensive in rural or decentralized populations in developing nations. As a result, research into more efficient and cost-effective water treatment systems is critical in order to attain a safe level [17]. Previously, efforts were focused on designing a treatment that might minimize costs by utilizing particular processes or activities such as adsorption, filtration, and precipitation. The adsorption technique is the most efficient, technically sophisticated, and well-known of these procedures, but the others have inherent limitations such as sludge development, poor efficiency, sensitive working conditions, and expensive disposal [18–31].

The goal of the present study is to look at the drinking water treatment and safety requirements in the Illu Abba Bor region. The drinking water samples were collected at three different villages Tulube, Seddo, and Serdo. They are analyzed for important water quality characteristics such as pH, turbidity, conductivity, total suspended solids (TSS), total dissolved solids (TDS), and heavy metal presence as per the WHO standards. The water was then treated with an adsorbent made from banana pseudostem. A homemade filter was constructed composed of filter media (sand, charcoal, and powder of banana pseudostem). The atomic absorption spectroscopy (AAS) was used to evaluate the purified water from the filter.

## 2. Materials and Methods

### 2.1. Narrative of the Study Area

**Figure 1:**
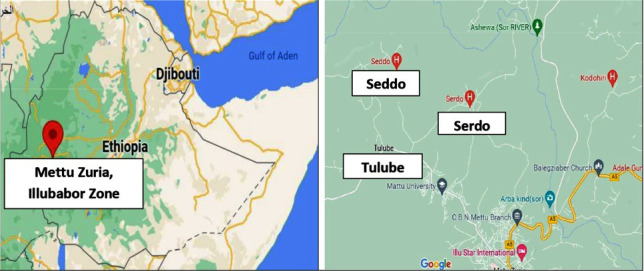
Representation of the study area.

The town of Mettu (8.2961°N, 35.5822°E) can be found in the Illu Abba Bor zone of the Oromia region of Ethiopia. It is about 550 km to the south-west of the capital city of Addis Ababa. The water used for drinking in the Illu Abba Bor zone comes either from the river Gore or the river Sor. In order to conduct an investigation on the physiochemical characteristics of the drinking water, three different villages located close to Mettu town, i.e., Tulube, Seddo, and Serdo, were selected and the samples were gathered.  Figure 1 represents the study area.

### 2.2. Analysis of Physiochemical Parameters

The cloudiness of water is referred to as turbidity. It is a measurement of light's ability to travel through water. Drinking water with excessive turbidity, or cloudiness, is both visually unappealing and potentially detrimental to one's health. In turbidity, pathogens can find food and shelter. The turbidity was measured using a turbidity meter (Wag-WT3020, Halma PLC Company). The pH of the water sample was measured using a digital pH meter (Hanna pH meter). Alkalinity in natural rivers is caused by the decomposition of CO_2_ in water. Carbonates and bicarbonates are formed and then dissociated to form hydroxyl ions. Water's alkalinity is its ability to neutralize acid. In water treatment methods and defluoridation operations, the alkalinity value is crucial for estimating the disinfection dose. The alkalinity was determined in accordance with APHA recommendations [32]. The general hardness of the water was mostly caused by the dissolved calcium salt and magnesium salt from the nearby ores. The hardness of the water will affect its taste. Water conductivity and total dissolved solids were measured in s/cm using a conductivity meter (WTW Inolab Cond 720). Water samples were tested for the content of calcium, magnesium, nitrates, phosphates, lead, copper, chromium, and zinc using a flame atomic absorption spectrometer (FAAS).

### 2.3. Preparation of Adsorbent from Banana Pseudostem

The samples of banana pseudostems were gathered from the local area. The samples that were gathered were then divided into smaller pieces that ranged in size from 5 to 10 mm. After that, it was properly cleaned with regular water in order to get rid of the mud and any other particles that were undesirable. After that, the material was exposed to the sun for seven days in order to evaporate any surface water. In the end, the materials that had been dried went through a grinding and sifting process. After that, it was collected and put away for later use in an area that was sealed off with an airtight zip lock cover.

### 2.4. Batch Experiments for Adsorption Process

In batch mode, 50 ml of the water sample is taken in a conical flask; the prepared adsorbent is added to it. The parameters, agitation duration (min), adsorbent dosage (g/L), and initial lead concentration in aqueous solution (mg/L) for each sample will be modified. The samples are agitated for a specified period. The lead removal (%) by adsorption is calculated as

(1) $$\begin{array}{c} Lead\;removal\left(\%\right)=\frac{\left(C_{0}-C_{t}\right)}{C_{0}}\times 100\text{,} \end{array}$$

where *C*_*t*_ indicates the lead ion concentration at different time at equilibrium condition and *C*_0_ indicates the initial lead ion concentration.

The adsorbed quantity on to the adsorbent surface was estimated using the equation below.

(2) $$\begin{array}{c} q_{e}=\frac{\left(C_{o}-C_{e}\right)V}{W}\text{,} \end{array}$$

where *C*_*o*_ and *C*_*e*_ (mg/L) are initial and equilibrium lead ion concentrations in aqueous solution, respectively, *W* (g) is the mass of adsorbent, and *V* (L) is the volume of the solution. The concentrations of the Pb (II) samples were determined using atomic absorption spectroscopy (AAS). All the experiments will be conducted in triplicate and the average values will be computed.

### 2.5. Adsorption Isotherms

The adsorption capacity was studied using Langmuir isotherm model. The experimental adsorption data were used with this model. Most of the literature studies show that the Langmuir adsorption isotherm is extensively used for adsorption that occurs at specific sites of a homogeneous adsorbent. Adsorption cannot take place once the adsorbate molecule occupies a site due to equilibrium being reached [10–12], and equation (3) describes the nonlinear form of the Langmuir model.

(3) $$\begin{array}{c} q_{e}=\frac{q_{\max}k_{L}C_{e}}{\left(1+k_{L}C_{e}\right)}\text{,} \end{array}$$

where *C*_*e*_ (in mg/L) denotes the concentration equilibrium of the lead ions in the solution, *q*_*e*_ (in mg/g) is the adsorption capacity, *q*_max_ (in mg/g) is the maximum adsorption capacity, and *k*_*L*_ (L/mg) is the Langmuir adsorption constant.

The favorability or unfavorability of the adsorption system can be predicted by the equilibrium parameter RL, which is a dimensionless constant that is an essential characteristic of the Langmuir model. The equilibrium parameter is determined by the following equation:

(4) $$\begin{array}{c} R_{L}=\frac{1}{1+{k_{L}C}_{O}}\text{.} \end{array}$$

This parameter suggests the type of isotherm, which may be irreversible (*R*_*L*_ = 0), favorable (0 < *R*_*L*_ < 1), or unfavorable (*R*_*L*_ > 1).

### 2.6. Experimental Setup—Treatment of Drinking Water

The adsorption column made with an acrylic material was used for conducting the water purification. The column is 59 cm tall and 10 cm wide.  Figure S1 provides the design of the water filter used in the experiment. Adsorption chambers are filled with filter media as adsorbent in the adsorption setup. The first layer is composed of banana pseudostem powder, the second of charcoal powder, and the third of sand particles. The treated water will pass through the top chamber, various layers, and finally the filtrate will reach the bottom chamber. The experiment was conducted and the filtrate was then analyzed using the AAS to determine the concentration of lead ions in the solution. Based on the (1), the percentage of removal can be calculated. Figures S2 and S3 provide visual representation of the adsorbent and water filter.

## 3. Results and Discussion

### 3.1. Study Area Assessment

Table 1 summarizes the physiochemical parameters of drinking water based on the findings of the examination of water samples obtained from three villages. The samples were collected from tap water of residential and commercial places of these three villages. The physiochemical parameters of the tested samples were mostly in agreement with the WHO standards [33]. However, the samples include considerable levels of lead, according to the findings. Lead is extremely toxic in its natural habitat and has a significant negative influence on the world's biodiversity. It is critical to remove lead and other heavy metals from water sources.

### 3.2. Batch Adsorption

#### 3.2.1. Effects of Contact Time

**Figure 2:**
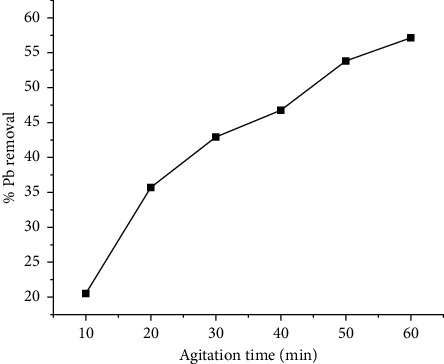
Effects of agitation time on lead removal.

In the process of adsorption, the contact time is an important parameter that offers an optimal value for the maximum amount of adsorption that may occur at the surface of the adsorbent [20, 21]. Therefore, in order to determine the effect that contact time has on the removal of lead ions, the experiments were carried out with varying time intervals ranging from 10–60 min, at an initial lead ion concentration of 30 mg/L and an adsorbent dosage of 0.5 g/L. The findings of the experiments are shown graphically in Figure 2. As can be seen from the figure, the Pb (II) concentration adsorbed on the banana stem increases with the prolonged time. The rate of the adsorption process initially depends on the mass transfer stage. The initial stage is characterized by high intense adsorption due to the availability of active sites on the surface of the adsorbents, therefore large concentration gradient activates the process [21]. Similar kind of phenomena observed in the present study too. The Pb (II) uptake was very fast initially, and the equilibrium attained at 60 min. Thus, in the following experiments, 60 min was selected as the optimal contact time. The fast adsorption suggests the process is mainly dominated by chemical adsorption rather than physical adsorption.

#### 3.2.2. Effects of Adsorbent Dosage

**Figure 3:**
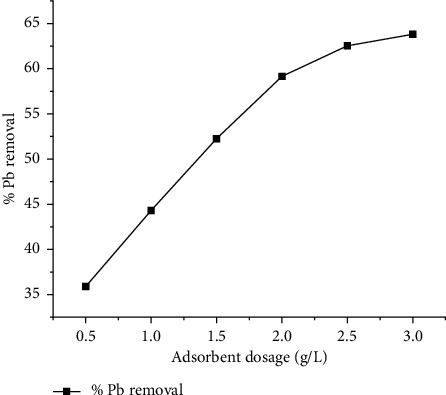
Effects of adsorbent dosage on lead removal.

The ideal adsorbent dosage is chosen carefully for effective and efficient removal of contaminants because adsorption is usually influenced by the dosage of adsorbent. The effect of dosage was studied using Pb (II) concentrations of 30 mg/L and the optimal contact time (60 min). The dosage amount varied from 0.5 to 3 g/L. After agitating, filtration was done and the concentration of heavy metal ions in the filtrate was determined using the AAS. The adsorbent dosage which adsorbed the maximum metal ions was considered to be the optimal dosage and was 0.25 g/L for all the metal ions (Figure 3). Further increase in the adsorbent dose thereafter did have much influence on the Pb (II) removal. This is due to overlapping of the adsorption sites because of overcrowding of adsorbent particles beyond the optimum dose and shielding of the adsorption sites [29, 30].

#### 3.2.3. Effects of Initial Concentration

**Figure 4:**
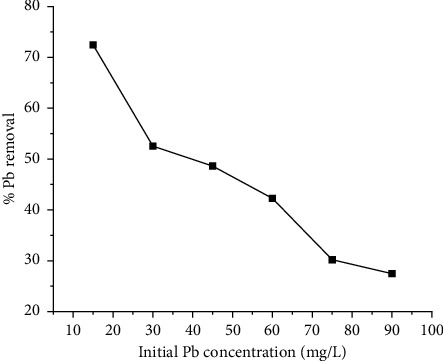
Effects of initial Pb concentration on lead removal.

Experiments were conducted with varying beginning ion concentrations, ranging from 15 to 90 mg/L, with increments of 15 mg/L, for a total of 60 min, with the adsorbent dose set at 2.5 g/L. This was done so that the impact of the initial ion concentration could be determined. The outcomes of the experiment are shown in graphical form in Figure 4. The investigations that were discussed above provide evidence that the adsorption percentage drops when the initial ion concentration is increased. The concentration of the Pb (II) with the highest uptake was taken to be optimal which was 10 mg/L.

#### 3.2.4. Adsorption Isotherm

**Figure 5:**
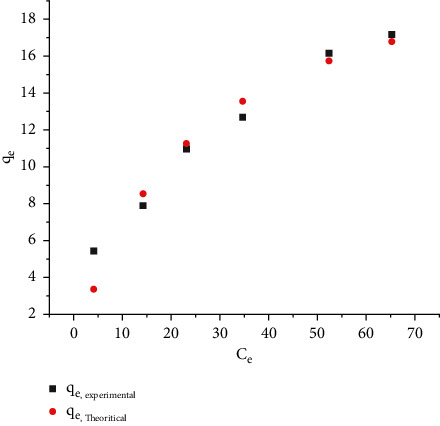
Langmuir isotherm.

The nonlinear regression analysis for the Langmuir isotherm is shown in Figure 5. The graph between *C*_*e*_ and *q*_*e*_ will be used to compute the adsorbent's maximum absorption capacity (*q*_max_) for the adsorption of Pb (II) ions from water samples. The Langmuir study yielded *q*_max_ and *k*_*L*_ values of 22.96 mg/g and 0.0415, respectively. *R*^2^ (regression coefficient) was determined to be 0.943. The equilibrium value *R*_*L*_ varies from 0.194 to 0.7, showing that adsorption is favorable.  Table 2 presents the removal uptake capacity of various adsorbents used for Pb removal.

### 3.3. Treatment of Drinking Water with Filter

**Figure 6:**
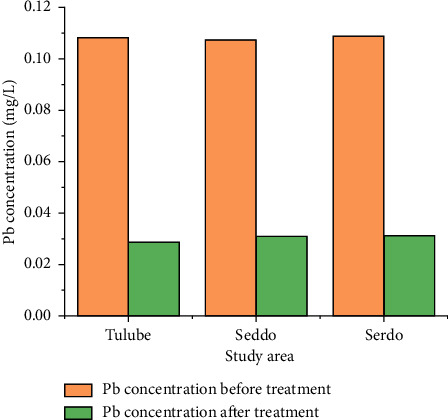
Adsorption of lead ions in drinking water modified homemade filter media containing banana pseudostem powder, charcoal powder, and sand particles (4 h treatment time).

The drinking water was purified using a modified homemade filter medium that was adjusted to include banana pseudostem powder, charcoal powder, and sand particles. The drinking water sample will be run through the filter beds, and the filtrate for each of the water samples obtained from the separate villages will be evaluated. The trials utilizing the modified handmade filter incorporating banana stem throughout time periods of 30 min, 1, 2, 3, 4, and 5 h revealed that the highest lead removal was reached with treatment duration of 4 h, and that equilibrium was achieved after that. A control experiment was carried out in which no adsorbent was used, i.e., just charcoal powder and sand particles were used as filter beds for water treatment. For all samples, the removal of lead with modified handmade filter media was greater than the standard filter media (the control). The control studies yielded a percentage of lead removal of 48.68%, 45.5%, and 45.8% for the three samples obtained from the various communities. The modified filter bed, which contained banana pseudostem, charcoal, and sand, respectively, removed 73.56%, 71.29%, and 71.38% of the lead. The conclusion of the experiment is given in graphical form in Figure 6. It demonstrates that, despite significant reductions in lead ion concentrations (about 70% in all three sites), the lead concentration remains greater than what the WHO considers safe. The results indicate that the modified handmade filter created for drinking water purification has a high potential for improvement.

Devi et al. [34] conducted a similar study on the removal of fluoride, arsenic, and coliform bacteria from drinking water using a modified handmade filter medium that incorporated crushed bricks. The residual amounts of fluoride, arsenic, and coliform bacteria were determined to be within the WHO's permissible criteria. Mengistie et al. [2] conducted an experiment to test the effect of a modified handmade filter on turbidity, fecal coliform, fluoride, as well as the filter's influence on temperature and pH. The study's findings suggest that filtering fluoridated raw water with the material employed in the study might remove considerable levels of physical, chemical, and biological contaminants. Abdel-Shafy et al. [4] investigated and examined the physicochemical properties of drinking water, as well as developing a recycling sand filter. The drinking water treatment approach that is already in use is an efficient means of purifying easily available fresh water.

## 4. Conclusions

Drinking water samples from three distinct communities were analyzed for quality in accordance with the WHO and EPA guidelines. The drinking water was judged to be safe, except for the presence of additional lead. Pb (II) ions were removed from drinking water using powdered banana stem as an adsorbent. The important findings from the batch trials are summarized below: the initial adsorbate concentration had a significant impact on how well the adsorption functioned (lead ions in solution). When the concentration of lead ions is higher to begin with, less lead is removed. Furthermore, both the amount of adsorbent used and the length of treatment contribute to an increase in adsorption capacity. According to the study's findings, lead ion might be eliminated by using a low-cost agricultural waste product known as banana pseudostem. According to the Langmuir isotherm analysis, the adsorbent's maximal absorption capacity was 22.96 mg/g. Furthermore, a homemade water filter comprised of sand, charcoal, and banana stem was employed as a water filter to remove lead ions. The adsorption took 4 h and effectively reduced the lead levels by 70%. Based on the research, we conclude that the homemade filter is successful in purifying drinking water; however, the concentration of lead ions need to be lower than the level set by the WHO. Therefore, it is vital to increase the effectiveness of the adsorption process by adjusting adsorption parameters such as treatment duration, the amount of adsorbent contained inside each column, and the particle size. In addition, the adsorbent has to be replaced every two days in order to keep it fresh. This will allow the adsorption process to be more effective while also preventing the growth of germs and other types of pollution.

## Figures and Tables

**Table 1:** Laboratory results for physiochemical water quality parameters of water sample.

S. no.	Parameters	Tulube	Seddo	Sardo	^#^WHO limit [33]	^*∗*^EPA [34]
1	pH	7.34	7.37	7.40	6.5–8.5	6.5–8.5
2	Conductivity (*μ*s/cm)	1800	1726	1650	2000	2000
3	TDS (ppm)	800	760	600	1000	500
4	Alkalinity (ppm)	300	280	290	500	600
5	Turbidity (NTU)	5.8	6.1	5.9	5	5–7
6	Chloride (mg/L)	180	190	185	260	250
7	Total hardness (ppm)	300	280	310	500	600
8	Calcium (mg/L)	30	35	32	75	-
9	Magnesium (mg/L)	30	40	30	150	-
10	Ammonia (mg/L)	0.5	0.6	0.7	1.5	-
11	Nitrate (mg/L)	25	28	30	50	45
12	Phosphate (mg/L)	30	25	25	50	45
13	Lead (ppm)	0.1082	0.1073	0.1087	0.01	0.005
14	Copper (ppm)	^$^BDL	0.0643	0.0661	1	1-1.3
15	Chromium (ppm)	0.0027	0.0023	0.0031	0.05	0.1
16	Zinc (ppm)	0.2102	0.2388	0.1333	1.2–2.5	5

^*∗*^EPA–Environmental Protection Agency. ^#^WHO–World Health Organization. ^$^BDL–below detection limit.

**Table 2:** Pb removal uptake capacity of various adsorbents.

S. no	Adsorbent	Uptake capacity (mg/g)	Reference
1	Lantana camara leaves'	3.5	[35]
2	Banana peel	7.97	[36]
3	Bael leaves (*Aegle marmelos*)	104	[37]
4	Mespilus germanica powder	4.53	[38]
5	Tribulus terrestris powder	3.26	
6	Pseudo banana stem	22.96	This study

## Data Availability

The data used to support the findings of this study are included within the tables and figures.

## Supplementary Materials

Supplementary Materials Figure S1 provides the design of the water filter used in the experiment. The column contains different chambers. The height of individual chambers is described in Figure S1. The first three chambers contain adsorbent materials. As described in Figure S1, layer 1 is filled with banana pseudostem powder, layer 2 is filled with charcoal powder, and layer 3 is filled with sand particles. The treated water will pass through the top chamber, then different layers, and finally the filtrate will reach the bottom chamber. The visual representation of pseudo-banana stems collected and processed is represented in Figure S2. Figure S3 represents the filtering materials and adsorption column.
