# Supplementary figures and images for: Physiochemical Analysis of Drinking Water and Treatment with a Homemade Filter: A Case Study of Illu Abba Bor Zone, Ethiopia
Source: Int J Anal Chem. 2022 Dec 31;2022:4333938. doi: 10.1155/2022/4333938 (PMC9825231; doi:10.1155/2022/4333938)

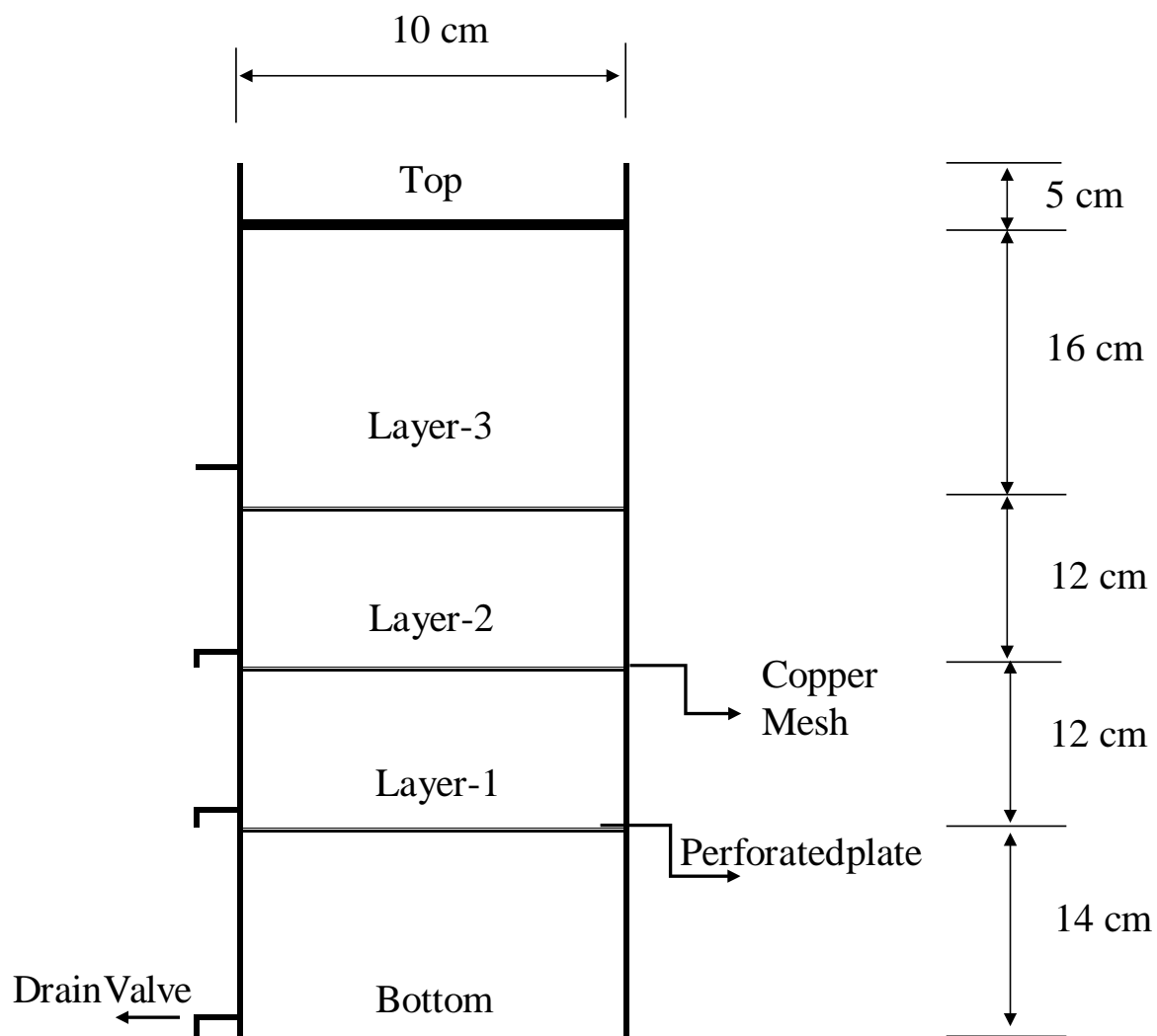

**Figure S1 Home Made Filter for Adsorption**

Supplement: Supplementary Materials — Figure S1 provides the design of the water filter used in the experiment. The column contains different chambers. The height of individual chambers is described in Figure S1. The first three chambers contain adsorbent materials. As described in Figure S1, layer 1 is filled with banana pseudostem powder, layer 2 is filled with charcoal powder, and layer 3 is filled with sand particles. The treated water will pass through the top chamber, then different layers, and finally the filtrate will reach the bottom chamber. The visual representation of pseudo-banana stems collected and processed is represented in Figure S2. Figure S3 represents the filtering materials and adsorption column. [file 4333938.f1.zip › Fig.S1.pdf]
